# Natural Mineral Water (*Hora*) Supplementation Improves Growth Performance and Carcass Characteristics of Sheep in Ethiopia

**DOI:** 10.1002/vms3.71008

**Published:** 2026-05-28

**Authors:** Ashenafi Miresa Kenea, Taye Tolemariam, Belay Duguma, Ellen S. Dierenfeld, Abebe Nigussie, Feyissa Begna, Sisay Bekele

**Affiliations:** ^1^ Department of Animal Science College of Agriculture and Veterinary Medicine Jimma University Jimma Oromia Ethiopia; ^2^ Department of Veterinary and Biosciences Faculty of Veterinary Medicine Ghent University Ghent Belgium; ^3^ Department of Natural Resource Management College of Agriculture and Veterinary Medicine Jimma University Jimma Oromia Ethiopia; ^4^ School of Veterinary Medicine College of Agriculture and Veterinary Medicine Jimma University Jimma Oromia Ethiopia

**Keywords:** carcass characteristics, growth performance, *hora*, nutrient intake, sheep

## Abstract

**Background:**

Natural mineral water (*hora*) is a mineral source widely available in Ethiopia. However, its specific impacts on sheep growth performance and carcass characteristics remain understudied.

**Objective:**

To evaluate the effect of *hora* supplementation on nutrient intake, growth performance and carcass characteristics of sheep.

**Methods:**

Twenty *Horro* breed sheep with an initial body weight (IBW) of 17.27 ± 0.71 kg were assigned to five treatment groups in a randomized complete block design (RCBD), blocked on the basis of their IBW. The treatments included control group (CON) fed basal diet only, and four treatment groups (H‐200, H‐300, H‐400, and H‐500) receiving 200, 300, 400, and 500 mL, of *hora* per day respectively. All sheep were fed grass hay and water ad libitum. Throughout the feeding trial data on dry matter (DM) and nutrient intake, body weight changes and various carcass traits were measured.

**Results:**

*Hora* supplementation significantly improved (*p* < 0.001) daily DM and nutrient intake. The H‐400 group exhibited the highest intake, with total DM intake reaching 0.99 kg/day (*p* < 0.001), whereas CON and H‐300 showed the lowest. Final body weight, live weight gain, average daily gain, and feed conversion efficiency were significantly higher (*p* < 0.001) in the H‐400 group. Polynomial contrasts revealed significant quadratic patterns (*p* < 0.05) for most intake and growth performance parameters. A significant linear trend (*p *< 0.001) was observed for empty body weight, hot carcass weight, and rib‐eye area. Dressing and saleable percentages remained unaffected by *hora* supplementation (*p* > 0.05). Total edible offal components showed a significant quadratic pattern (*p* = 0.001), whereas total non‐edible offal showed a significant linear trend (*p* = 0.002).

**Conclusions:**

Supplementation with 400 mL/day of *hora* is the optimal dose for significantly improving sheep growth performance, nutrient intake, and carcass characteristics. Overall, although *hora* provides clear benefits, its effects are dose‐dependent, with potential diminishing returns beyond the 400 mL/day level.

## Graphical abstract

This study investigates the effect of natural mineral water (*hora*) supplementation on sheep performance in Ethiopia. Optimal level of *hora* supplementation significantly improved nutrient intake, growth performance and carcass characteristics of sheep.

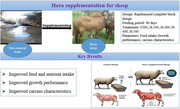

## 1 Introduction

Sheep require balanced diets that include all necessary nutrients, including minerals, for optimal growth, production, reproduction and immunity (Simões et al. 2021). Among various nutritional strategies, mineral supplementation has emerged as a critical component in improving the overall well‐being and performance of sheep (Stewart et al. 2021; Hession et al. 2022). Mineral imbalance and deficiencies are recognized as major nutritional constraints to sheep productivity (Fadlalla 2022). Poor growth performance in sheep is frequently associated with deficiencies in essential trace minerals, particularly cobalt, copper, iodine, manganese and selenium (Helmer et al. 2021). In many developing regions, such as Ethiopia, basal diets including crop residues and native grasses often fail to meet the daily essential mineral requirement, leading to significant health issues and economic loss (Dermauw et al. 2013; Saha and Pathak 2021).

Although commercial mineral blocks and premixes are available, their high cost and inconsistent availability often make them inaccessible to smallholder farmers. Consequently, local farmers have traditionally relied on natural mineral water, known as *hora*, as a cost‐effective and readily available alternative (Zeleke et al. 2016; Tilahun and Mengistu 2019). Although previous studies have explored the effect of organic and inorganic mineral supplements (Lalhriatpuii et al. 2024; Zhang et al. 2024) and some natural sources like salt licks (Panichev et al. 2017; Tawa et al. 2022; Wang et al. 2023), the importance of *hora*, a readily available and locally sourced mineral water in Ethiopia, has not been extensively investigated. Additionally, the specific impacts of *hora* on growth performance, nutrient utilization and carcass characteristics of sheep remain largely unexplored. Given that the availability of *hora* and the prevalence of mineral deficiency in sheep, its role as a mineral source and its impact on sheep performance is essential for optimizing its contribution to sheep nutrition. Thus, the present study was aimed to evaluate the effect of *hora* supplementation on growth performance, nutrient intake and carcass characteristics of sheep.

## 2 Materials and Methods

### 2.1 Description of the Study Site

The experiment was conducted at Jimma University College of Agriculture and Veterinary Medicine small ruminant farm between April and June 2024. The experimental site is located at 7° 41′ 06″ N latitude and 36° 49′ 52″ E longitude coordinates and an elevation of 1746 m above sea level. The maximum average monthly temperature varies from 24.7°C to 30.0°C, with an overall average temperature of 27.6°C, whereas the minimum monthly temperature varies between 13.8°C and 7.7°C, with a cumulative average of 11.7°C (EMI 2024). The experimental site receives an average annual rainfall of 1529 mm.

### 2.2 *Hora* Collection

Experimental water *(hora)*, sourced from spring water, was collected from Dabo district, Buno Bedele Zone and transported to experimental site. Forty clean plastic jars, with a capacity of 20 L, were filled with *hora* and transported to experimental site. The total volume of *hora* transported to the experimental site was calculated on the basis of the specific volume set to each treatment group, taking into account potential loss during transport and supplementation to the sheep.

### 2.3 Experimental Animals and Their Management

Twenty weaned *Horro* breed male sheep with an initial body weight (IBW) of 17.27 ± 0.71 kg were purchased from the local market. Upon arrival, the sheep were ear‐tagged for identification and housed individually in separate pens (1.5 × 1 m^2^). Prior to experimental data collection, each sheep was allowed 15 days of adaptation to the experimental diet, vaccinated against shoat pox, and treated against internal and external parasites using Albendazole (AlbenNat 300 mg, China), and Ivermectin (Ivertong 10, China), respectively.

### 2.4 Experimental Design and Treatments

The experiment was used a randomized complete block design (RCBD), and the sheep were blocked into five groups of four animals each, on the basis of their IBWs. Treatments were then randomly allocated to the animals within each block. The experimental diets were: Treatment 1 (CON): a basal diet only (control). Treatments 2–5 (H‐200, H‐300, H‐400, and H‐500): Basal diet supplemented with *hora* mineral water at 200, 300, 400, and 500 mL/day, respectively.

### 2.5 Diet Formulation and Feeding Protocol

The diet formulation targeted a concentrate to roughage ratio of 60:40 on a dry matter (DM) basis to meet the daily nutrient requirement for growing lamb (NRC 2007). The concentrate mixture was composed of maize (38%), Niger seed cake (*Guizotia abyssinica*) (27%), wheat bran (32%), calcium carbonate (2%), and common salt (1%). The chemical compositions of the concentrate feed ingredients, grass hay and the mineral concentrations of the *hora* supplement are shown in Tables 1 and 2, respectively. To implement the feeding protocol, a fixed daily concentrate allowance was calculated as 60% of the target total dry matter intake (DMI), with the total DMI estimated at 2.5% of the sheep's body weight (BW × 2.5% × 60% on a DM basis), a value consistent with established guidelines for the maintenance and moderate growth of indigenous tropical sheep (NRC 2007; Sileshi et al. 2021). This fixed amount of concentrate was provided in two equal proportions per day at 20:00, 06:00 h. Grass hay and water were provided ad libitum to allow the animals to consume roughage freely, ensuring that their remaining fibre, and nutrient requirements (expected to be 40% or more of total intake) were met. The amount of feed offered was adjusted weekly based on the animals' body weight changes. To ensure complete consumption of the allocated *hora* mineral water, each sheep was provided with an individual bucket with its designated volume. Access to regular water was restricted until each sheep had consumed the provided volume of *hora*, and careful observation confirmed that all animals readily consumed their allocated amount.

**TABLE 1:** Chemical composition (%DM basis) and mineral concentration of feed ingredients.

Feed ingredients	Proximate composition (%)								
	DM	Ash	OM	CP	ADL	ADF	NDF	EE	ME
Niger seed cake	92.1	8.7	91.3	30.9	12.4	30.7	34.8	9.0	11.5
Wheat bran	88.0	6.1	83.9	16.6	5.1	15.4	43.3	3.8	11.7
Maize	91.1	4.1	95.9	7.6	1.8	3.3	16.4	5.2	13.6
Grass hay	91.0	9.2	90.8	10.8	18.8	43.4	63.4	3.6	8.8
Concentrate mixture^a^	90.6	8.8	91.2	16.5	5.7	14.5	29.5	5.6	12.0

Feed ingredients	Macro‐minerals (g/kg DM)						Micro‐minerals (mg/kg DM)					
	Ca	Mg	Na	K	P	S	Mo	Fe	Mn	Cu	Zn	Se
Niger seed cake	7.1	5.5	0.1	12.8	10.9	2.5	0.5	2148.0	128.0	24.0	65.0	2.1
Wheat bran	1.4	4.5	0.1	13.7	11.1	2.1	0.5	155.0	114.0	13.0	89.0	0.1
Maize	0.4	1.3	0.5	3.6	2.9	0.8	0.14	116.0	14.0	10.0	24.0	0.1
Grass hay	0.9	0.2	0.1	4.1	0.4	2.5	0.2	250.0	41.1	1.2	8.2	0.2

Abbreviations: ADF, acid detergent fibre; ADL, acid detergent lignin; CP, crude protein; DM, dry matter; EE, ether extract; ME, metabolizable energy; NDF, neutral detergent fibre; OM, organic matter.

^a^ Concentrate mixture values were calculated based on the proportional inclusion of each ingredient and its corresponding proximate analysis.

**TABLE 2:** The mineral concentrations of the *hora* used as experimental diet for sheep.

Macro‐minerals (g/L)							Micro‐minerals (mg/L)					
Treatments	Ca	Mg	Na	K	P	S	Mo	Fe	Mn	Cu	Zn	Se
CON	—	—	—	—	—	—	—	—	—	—	—	—
H‐200	0.09	0.06	1.10	0.05	0.01	0.03	0.29	3.17	0.11	0.09	0.26	0.06
H‐300	0.15	0.09	1.65	0.08	0.01	0.05	0.44	4.76	0.16	0.14	0.39	0.09
H‐400	0.19	0.13	2.21	0.11	0.01	0.07	0.59	6.34	0.22	0.18	0.53	0.13
H‐500	0.24	0.16	2.76	0.13	0.01	0.09	0.73	7.93	0.27	0.23	0.67	0.16
*Hora*/L	0.49	0.32	5.51	0.26	0.03	0.17	1.47	15.85	0.54	0.46	1.32	0.32

*Note*: The mineral concentrations in H‐200, H‐300, H‐400, and H‐500 were calculated based on the laboratory analysis of *hora* mineral water, taking into account the specific inclusion levels of each treatment.

### 2.6 Measurements and Laboratory Analysis

#### 2.6.1 Chemical Composition of Feed Samples

The feed DM, ash, crude protein (CP), ether extract (EE), neutral detergent fibre (NDF), acid detergent fibre (ADF), and acid detergent lignin (ADL) were analysed according to AOAC official methods of 930.15, 942.05, 990.03, 973.18 and 973.18, respectively, outlined in the AOAC (2023) procedure. Nitrogen‐free extract (NFE) was calculated as: NFE = 100 − (CP + EE + Ash + Crude fibre [CF]). The metabolizable energy (ME) value was calculated using digestibility coefficients for ruminant obtained from the INRA‐CIRAD‐AFZ feed tables (Sauvant et al. 2004). The formula used to calculate ME was: ME (MJ/kg DM) = (15.2 × dCP + 34.2 × dEE + 12.8 × dCF + 15.9 × dNFE)/1000, where dCP, dCFat, dCF, and dNFE represent the digestible CP, EE, CF, and NFE, respectively. The concentrations of macro and trace minerals (calcium [Ca], magnesium [Mg], sodium [Na], potassium [K], phosphorus [P], sulphur [S], molybdenum [Mo], iron [Fe], manganese [Mn], copper [Cu], zinc [Zn], and selenium [Se]) in basal diet and *hora* were analytically determined using the ICP‐OES (FSH‐12‐2010; Arcos‐SOP‐ICP‐OES, the Netherlands) instrument following the standard procedure for mineral analysis.

#### 2.6.2 DM, Nutrient Intake and Body Weight Change

The feeding trial lasted for 90 days. Daily DMI for both the concentrate and grass hay was determined by calculating the difference between feed offered and feed refused, measured daily and adjusted for the DM content of the respective feeds. The total daily DMI (TDM) was calculated as the sum of concentrate DMI and hay DMI. The intake values of organic matter (OM), CP, NDF, ADF, ADL, and ME were determined by multiplying feed intake by the corresponding percentage of each component in the feed as suggested by Balehegn et al. (2014). The IBW was calculated by taking the average of two consecutive measurements. Subsequent body weights were recorded at 10‐day intervals, and the final body weight (FBW) was measured at the end of the feeding trial. Live weight gain (LWG) was calculated as the difference between IBW and FBW. Average daily body weight gain (ADG) was calculated by subtracting the IBW from the FBW and dividing by the number of feeding Days (90). The feed conversion efficiency (FCE) was determined by dividing daily weight gain by the daily amount of feed consumed. The average daily mineral intake from the basal diet was calculated on the basis of the daily intake of grass hay and concentrate mixture by each sheep. The daily absolute intake of each mineral from *hora* water for each treatment group was calculated by multiplying the mineral concentration in *hora* water by the daily volume of *hora* supplementation (0.2, 0.3, 0.4 and 0.5 L for H‐200, H‐300, H‐400 and H‐500 groups, respectively). The total daily absolute mineral intake for each treatment was then determined by summing the calculated mineral intake from the basal diet and the mineral intake from the *hora* water supplementation.

#### 2.6.3 Carcass Traits Evaluation

At the end of the experimental period, the sheep were subjected to overnight fasting, with free access to water for carcass characteristic evaluation. Animals were slaughtered according to the standard commercial procedures after weight was recorded just before slaughter to obtain the fasted live weight (FLW). At slaughter, the weights of edible offal (blood, heart, liver, kidney with fat, tongue, stomach, large and small intestine, tail, and oesophagus) and non‐edible offal components (head without tongue, skin with feet, penis, lung with trachea, spleen, gall bladder with bile, urinary bladder, pancreas, and testicle) were recorded. We categorized edible and non‐edible offal components according to local people meat consumption habits. The weight of the digestive tract was calculated as the difference between the weights of full and empty digestive tracts. Empty body weight (EBW) was obtained by subtracting the full gut content from FLW. Rib‐eye area (REA) muscle was calculated by measuring the cross‐sectional area of the longissimus dorsi muscle between the 12th and 13th ribs after cutting perpendicular to the backbone. Dressing percentage (DP) was calculated as the ratio of hot carcass weight (HCW) to FLW. The total edible offal (TEO) and total non‐edible offal (TNEO) were calculated as the sum of the TEO components and TNEO components, respectively. Saleable percentage (SP) was calculated as 100 × (FLW − head without tongues − gut contents − pancreas − spleen)/FLW as suggested by Balehegn et al. (2014).

### 2.7 Statistical Analysis

Data on DMI, weight gain, and carcass parameters were subjected to Analysis of Covariance (ANCOVA) using R statistical software, version 4.4.1 (R Core Team 2024). Although the experiment followed an RCBD, IBW was included as a continuous covariate to statistically adjust the treatment means for pre‐existing differences in initial weight. Prior to the final analysis, the assumption of homogeneity of regression slopes was verified by testing the interaction between the treatment and the IBW covariate. As this interaction was non‐significant (*p* > 0.05), it was removed from the final model, and a common slope was assumed for all treatments. Polynomial contrasts (linear and quadratic) were applied to evaluate the dose‐response relationship of *hora* levels. The ANCOVA model used was as follows:

$$Yijk=\mu +\mathrm{Trt}i+\mathrm{Rep}j+\beta \left(\mathrm{IBWijk}-\bar{\mathrm{IBW}}\right)+\in ijk$$

where *Y_ijk_* is the response variable and *μ* is the overall mean. Trt*_i_* is the fixed effect of the *i*th treatment (dietary level). Rep*_j_* is the fixed effect of the *j*th replicate (block effect). *β* is the regression coefficient representing the relationship between the response variable and IBW. *β*(IBW*_ijk_* − $\bar{\mathrm{IBW}}$) represents the deviation of the *k*th animal's initial body weight from the overall mean initial body weight. *ϵ_ijk_* is the random error associated with each observation.

## 3 Results

### 3.1 DM and Nutrient Intake

The inclusion of *hora* led to marked improvements (*p* < 0.001) in daily DM and nutrient intake compared to the control group (Table 3). Notably, the H‐400 treatment group achieved the highest total DMI at 0.99 kg/day. In contrast, both the CON and H‐300 groups exhibited the lowest intake levels. The daily ME intake was significantly higher (*p* < 0.001) in the H‐400 group (9.77 MJ/day) compared to the CON, H‐200 and H‐300 group, showing a significant (*p* = 0.026) quadratic trend. Polynomial contrasts revealed that *hora* supplementation significantly increased all intake parameters following a quadratic pattern (*p* < 0.05) with the exception of OM intake which was showed only a significant (*p* < 0.001) linear response.

**TABLE 3:** Daily dry matter and nutrient intake (%DM basis) of experimental sheep.

Intake parameter (kg/day)	Treatments					RSE	Treatment *p* value	Contrast *p* value	
	CON	H‐200	H‐300	H‐400	H‐500			Linear	Quadratic
Concentrate	0.39^bc^	0.38^bc^	0.36^c^	0.45^a^	0.42^ab^	0.02	<0.001	<0.001	0.024
Hay	0.49^bc^	0.49^bc^	0.47^c^	0.54^a^	0.52^ab^	0.01	<0.001	<0.001	0.041
TDM	0.89^bc^	0.87^bc^	0.83^c^	0.99^a^	0.94^ab^	0.03	<0.001	<0.001	0.027
OM	0.77^c^	0.81^bc^	0.76^c^	0.92^a^	0.87^ab^	0.03	<0.001	<0.001	0.645
CP	0.15^bc^	0.15^bc^	0.14^c^	0.17^a^	0.16^ab^	0.01	<0.001	<0.001	0.024
NDF	0.36^bc^	0.35^bc^	0.34^c^	0.40^a^	0.38^ab^	0.01	<0.001	<0.001	0.024
ADF	0.20^bc^	0.20^bc^	0.19^c^	0.23^a^	0.21^ab^	0.01	<0.001	<0.001	0.024
ADL	0.08^bc^	0.08^bc^	0.07^c^	0.09^a^	0.08^ab^	0.01	<0.001	<0.001	0.024
ME (MJ/day)	8.68^bc^	8.49^bc^	8.07^c^	9.77^a^	9.22^ab^	0.29	<0.001	<0.001	0.026

*Note*: CON: Control (without *hora* supplementation); H‐200: 200 mL of *hora* supplement; H‐300: 300 mL of *hora* supplement; H‐400: 400 mL of *hora* supplement; H‐500: 500 mL of *hora* supplement. Estimated marginal means across rows with different superscripts (a–c) are significantly different (*p* < 0.05).

Abbreviations: ADF, acid detergent fibre; ADL, acid detergent lignin; CP, crude protein; ME (MJ/day): metabolizable energy (Mega joule); NDF, neutral detergent fibre; OM, organic matter; RSE, residual standard error; TDM, total dry matter.

### 3.2 Daily Mineral Intakes

The daily intake of macro and micro‐minerals showed varied patterns across the treatment groups (Table 4). The CON, H‐200, and H‐300 groups exhibited deficiencies in key minerals, specifically Ca, P, S, and Cu, failing to meet the recommended daily requirements for growing lambs. Interestingly, in the H‐300 several mineral (including Ca, P, K, S, Zn and Cu) intakes were also insufficient. Conversely, although Na, K, and Mg intakes across all groups were within adequate ranges, *hora* supplementation at higher levels led to intakes of Fe and Se that exceeded the maximum tolerable limits (MTLs) in some groups. Overall, non‐linear patterns were observed for several minerals, with some groups, notably H‐300, showing intakes that sometimes fell below control levels.

**TABLE 4:** Daily mineral intakes from *hora* and basal diet for sheep across treatment groups.

Minerals	CON	H‐200	H‐300	H‐400	H‐500	Daily requirement^a^	MTL^b^
Calcium (g/day)	**3.67**	**4.07**	**2.96**	5.13	**4.50**	5.0–15.0	13.40
Sodium (g/day)	1.56	2.78	2.84	4.27	4.54	0.8–1.8	53.70
Phosphorus (g/day)	**3.17**	**3.43**	**2.43**	4.23	3.67	3.5–7.0	5.40
Potassium (g/day)	5.42	5.96	**4.30**	7.67	6.57	5.0–20.0	33.60
Magnesium (g/day)	1.53	1.72	1.27	2.19	1.94	1.2–1.1.8	4.00
Sulphur (g/day)	**0.56**	**0.65**	**0.49**	1.88	1.77	1.4–1.6	2.70
Manganese (mg/day)	46.70	51.12	36.63	65.95	56.11	20.0–40.0	1342.80
Zinc (mg/day)	24.40	26.68	**19.16**	33.54	29.06	20.0–33.0	503.50
Copper (mg/day)	**6.14**	**6.72**	**4.84**	8.40	7.32	7.0–11.0	16.80
Iron (mg/day)	265.56	290.28	208.47	**363.48**	315.76	30.0–50.0	335.70
Selenium (mg/day)	0.26	**0.35**	0.30	**0.48**	**0.46**	0.1–0.2	0.30
Molybdenum (mg/day)	0.18	0.49	0.58	0.83	0.95	0.1–0.5	6.70

*Note*: CON: Control (without *hora* supplementation); H‐200: 200 mL of *hora* supplement; H‐300: 300 mL of *hora* supplement; H‐400: 400 mL of *hora* supplement; H‐500: 500 mL of *hora* supplement. Bold values indicate intakes that are either below the daily requirement or above the MTL.

^a^ Daily mineral requirements for growing sheep according to NRC (2007).

^b^ MTL: maximum tolerable limit for growing sheep according to NRC (2007).

### 3.3 Growth Performance and FCE

*Hora* supplementation had a highly significant impact (*p* < 0.001) on all growth performance parameters (Table 5). Sheep supplemented with H‐400 exhibited the higher FBW, LWG and ADG compared to other groups. The CON and H‐300 group consistently showed the lowest growth performance. Furthermore, sheep supplemented with H‐400 followed by H‐500 showed the higher FCE. Polynomial contrast analysis revealed that the responses for FBW, LWG, and ADG followed a significant quadratic pattern (*p* = 0.001).

**TABLE 5:** Growth performance and feed conversion efficiency.

Intake parameters (kg/day)	Treatments					RSE	Treatment *p* value	Contrast *p* value	
	CON	H‐200	H‐300	H‐400	H‐500			Linear	Quadratic
IBW (kg)	17.18^ab^	17.88^a^	17.01^b^	17.95^a^	16.32^b^	0.51	<0.001	N/A	N/A
FBW (kg)	24.10^bc^	24.80^b^	23.50^c^	29.80^a^	24.70^b^	0.36	<0.001	<0.001	0.001
LWG (kg)	6.83^bc^	7.56^b^	6.21^c^	12.58^a^	7.45^b^	0.36	<0.001	<0.001	0.001
ADG (g/day)	75.90^bc^	84.00^b^	69.00^c^	139.70^a^	82.70^b^	4.04	<0.001	<0.001	0.001
FCE	0.10^b^	0.11^b^	0.12^b^	0.15^a^	0.12^ab^	0.01	<0.001	<0.017	0.071

*Note*: CON: Control (without *hora* supplementation); H‐200: 200 mL of *hora* supplement; H‐300: 300 mL of *hora* supplement; H‐400: 400 mL of *hora* supplement; H‐500: 500 mL of *hora* supplement. Estimated marginal means across rows with different superscripts (a–c) are significantly different (*p* < 0.05).

Abbreviations: ADG, average daily gain; FBW, final body weight; FCE, feed conversion efficiency; IBW, initial body weight; LWG, live weight gain; N/A, not applicable; RSE, residual standard error.

### 3.4 Carcass Characteristics

The carcass characteristics of experimental sheep are presented in Table 6. *Hora* supplementation significantly influenced EBW, HCW and REA (*p* < 0.001), with the highest values observed in sheep supplemented with H‐400. A polynomial contrast analysis revealed a significant linear response (*p* < 0.001) for EBW, HCW and REA. Although the overall treatment effect showed significant (*p* = 0.047) difference for DP, subsequent post hoc analysis revealed no significant pairwise differences among the treatment groups. In contrast, SP showed no significant differences among treatments (*p* = 0.849).

**TABLE 6:** Carcass characteristics of the experimental sheep.

Parameters	Treatments					RSE	Treatment *p* value	Contrast *p* value	
	CON	H‐200	H‐300	H‐400	H‐500			Linear	Quadratic
EBW (kg)	18.10^b^	17.00^bc^	15.60^c^	22.60^a^	18.30^b^	0.76	<0.001	<0.001	0.181
HCW (kg)	11.28^b^	11.55^b^	9.51^c^	14.62^a^	11.28^b^	0.02	<0.001	<0.001	0.847
DP (%)	47.00	47.80	43.70	51.00	44.80	1.75	0.047	0.653	0.406
SP (%)	79.10	79.40	79.30	79.70	79.90	1.37	0.849	0.379	0.900
REA (cm^2^)	7.90^b^	8.09^b^	6.65^c^	10.24^a^	7.89^b^	1.08	<0.001	<0.001	0.847

*Note*: CON: control (without *hora* supplementation); H‐200: 200 mL of *hora* supplement; H‐300: 300 mL of *hora* supplement; H‐400: 400 mL of *hora* supplement; H‐500: 500 mL of *hora* supplement. Estimated marginal means across rows with different superscripts (a–c) are significantly different (*p* < 0.05).

Abbreviations: DP, dressing percentage; EBW, empty body weight; HCW, hot carcass weight; REA, rib‐eye area; RSE: residual standard error; SP, saleable percentage.

### 3.5 Edible Offal Components

*Hora* supplementation significantly influenced the weights of most edible offal components (TEO, blood, liver, kidney, tail, stomach and intestine), with the highest weights generally observed at the H‐400 level (Table 7). The weights of the tail showed a significant linear increase (*p* < 0.001) with increasing *hora* levels. In contrast, a significant quadratic pattern was observed for the weights of blood (*p* = 0.007), liver (*p* < 0.001), tongue (*p* = 0.035), intestine (*p* = 0.021) and TEO (*p* = 0.001). Heart (*p* = 0.007), kidney (*p* < 0.001), oesophagus (*p* = 0.027) and stomach (*p* < 0.001) weights all showed a significant overall treatment effect but did not exhibit clear polynomial trends.

**TABLE 7:** Edible offal components of experimental sheep.

Parameters	Treatments					RSE	Treatment *p* value	Contrast *p* value	
	CON	H‐200	H‐300	H‐400	H‐500			Linear	Quadratic
Blood (kg)	1.07^bc^	1.03^bc^	0.78^c^	1.42^a^	1.22^ab^	0.09	<0.001	<0.001	0.007
Heart (kg)	0.12^a^	0.13^a^	0.09^b^	0.12^a^	0.11^ab^	0.01	0.007	0.316	0.098
Liver (kg)	0.37^c^	0.41^b^	0.36^c^	0.44^a^	0.37^c^	0.01	<0.001	0.095	<0.001
Kidney (kg)	0.10^ed^	0.16^a^	0.09^cd^	0.13^bc^	0.11^bc^	0.01	<0.001	0.096	0.082
Tail (kg)	0.53^c^	0.53^c^	0.56^bc^	0.73^a^	0.69^ab^	0.05	<0.001	<0.001	0.374
Tongue (g)	79.00	83.40	80.50	85.10	80.00	2.56	0.108	0.389	0.035
Oesophagus (g)	39.70	41.60	33.60	43.80	45.70	4.71	0.027	0.080	0.062
Stomach (kg)	0.73^bc^	0.79^ab^	0.64^c^	0.87^a^	0.75^ab^	0.03	<0.001	0.085	0.793
Large and small intestine (kg)	0.87^c^	0.99^b^	0.87^bc^	1.12^a^	0.95^bc^	0.02	<0.001	0.003	0.021
Total edible offal (kg)	2.31^c^	2.39^c^	1.99^d^	2.97^a^	2.63^b^	0.03	<0.001	<0.001	0.001

*Note*: CON: control (without *hora* supplementation); H‐200: 200 mL of *hora* supplement; H‐300: 300 mL of *hora* supplement; H‐400: 400 mL of *hora* supplement; H‐500: 500 mL of *hora* supplement. Estimated marginal means across rows with different superscripts (a–c) are significantly different (*p* < 0.05).

Abbreviation: RSE, residual standard error.

### 3.6 Non‐Edible Offal Components

*Hora* supplementation significantly impacted all non‐edible offal components (Table 8). The H‐400 group exhibited the highest weights of TNEO, head without tongues, skin with feet and testicles. Polynomial contrasts revealed a significant quadratic pattern (*p* < 0.05) for the weights of the penis, lung with trachea, urinary bladder and pancreas. Conversely, a significant linear trend was observed for the head without tongue, skin with feet, testicle, spleen and TNEO (*p *< 0.05), with no significant quadratic trends. The gallbladder with bile showed a significant overall treatment effect (*p* = 0.013) but did not exhibit a clear polynomial trend.

**TABLE 8:** Non‐edible offal components of experimental sheep.

Parameters	Treatments					RSE	Treatment *p* value	Contrast	
	CON	H‐200	H‐300	H‐400	H‐500			Linear	Quadratic
Head without tongue (kg)	1.29^b^	1.33^b^	1.20^b^	1.78^a^	1.28^b^	0.10	<0.001	0.024	0.089
Skin with feet (kg)	3.08^b^	3.02^b^	2.73^b^	3.73^a^	3.19^ab^	0.17	<0.001	0.006	0.336
Penis (g)	78.20^a^	58.30^b^	48.20^b^	47.50^b^	45.70^b^	5.17	<0.001	<0.001	<0.001
Testicle (kg)	0.28^b^	0.31^b^	0.27^ab^	0.36^a^	0.30^ab^	0.02	0.024	0.006	0.116
Lung with trachea (kg)	0.36^a^	0.34^ab^	0.22^c^	0.35^ab^	0.31^b^	0.02	<0.001	0.007	<0.001
Spleen (g)	45.00^bc^	49.40^ab^	38.80^c^	55.00^a^	51.50^a^	2.69	<0.001	0.001	0.053
Gallbladder with bile (g)	13.34^ab^	20.75^a^	6.08^c^	6.69^c^	14.14^ab^	5.35	0.013	0.170	0.154
Urinary bladder (g)	10.00^b^	65.80^a^	32.30^ab^	13.50^b^	19.00^b^	9.07	<0.001	0.036	<0.001
Pancreas (g)	22.00^b^	35.70^a^	30.40^a^	33.30^a^	31.90^a^	2.26	<0.001	<0.001	<0.001
Total non‐edible offal (kg)	6.63^bc^	6.76^bc^	6.08^c^	7.77^a^	6.78^b^	0.21	<0.001	0.002	0.732

*Note*: CON: control (without *hora* supplementation); H‐200: 200 mL of *hora* supplement; H‐300: 300 mL of *hora* supplement; H‐400: 400 mL of *hora* supplement; H‐500: 500 mL of *hora* supplement. Estimated marginal means across rows with different superscripts (a–c) are significantly different (*p* < 0.05).

Abbreviation: RSE, residual standard error.

## 4 Discussion

### 4.1 DM and Nutrient Intake

The increased DM and nutrient intake observed in sheep supplemented with *hora* directly supported the improved growth performance and carcass yield. The daily intake of TDM, CP, and ME in the H‐400 and H‐500 groups was comparable to the minimum requirements for growing lamb established by NRC (2007). Although the CP intake in the CON group (0.14 kg/day) fell slightly below the minimum NRC requirement for growing sheep (150–250 g/day), the supplemented groups met and exceeded this benchmark. The effectiveness of *hora* in stimulating appetite is primarily attributed to its highly bioavailable mineral content such as Na, Cu, and Zn. Hossein Yazdi et al. (2019) similarly reported that sheep fed different levels of mineral‐vitamin supplements exhibited increased DMI. As a liquid supplement, *hora's* minerals are less susceptible to binding by dietary antagonists, such as phytates and oxalates, than those in solid feed (Humer et al. 2015; Zhang et al. 2022). This leads to enhanced absorption and metabolic function, potentially increasing mineral deposition in tissues and reducing excretion, thereby improving feed utilization and overall animal performance (Grešáková et al. 2021; Byrne and Murphy 2022).

The observed significant quadratic trends across most intake variables confirm that *hora* supplementation exerted a non‐linear biphasic effect; it stimulated DMI up to an optimal point (H‐400), after which the benefit begins to decline. It is noteworthy that while the overall trend was positive, a localized reduction in DMI was observed in the H‐300 group compared to the H‐200 group. This pattern strongly aligns with the principle of hormesis, a biological phenomenon where low‐dose exposure to a stimulant (minerals) yields beneficial physiological effects, whereas high‐dose exposure becomes inhibitory (Calabrese 2014; Bondy 2003). In this study, *hora* functioned as a hormetic agent, stimulating appetite and nutrient intake up to an optimal threshold of 400 mL/day. Beyond this point, the response transitioned into an inhibitory phase, likely due to metabolic stress from elevated mineral loads (Suttle 2022).

The lower nutrient intake in the H‐300 group directly accounts for their reduced growth performance relative to the other supplemented groups. Moreover, the superior performance at H‐400 indicates a balanced mineral profile that optimizes rumen microbial activity and fermentation efficiency (Zhang et al. 2024). Conversely, the decline at H‐500 represents the toxic arm of the hormetic curve. Excessive intake of specific minerals, particularly Fe and Se, can trigger physiological feedback mechanisms that limit further ingestion to prevent systemic toxicity (Suttle 2022). This quadratic response is consistent with recent findings in dietary additive research, where exceeding optimal supplementation levels leads to diminishing returns and impaired growth performance (Al‐Homidan et al. 2022; Katongole and Yan 2020).

### 4.2 Daily Mineral Intake

The basal diet and lower levels of *hora* supplementation resulted in deficiencies of several key macro‐minerals (Ca, P and S) and Cu. This finding is consistent with challenge faced in tropical subtropical regions, where basal diets often lack sufficient mineral content (Arthington and Ranches 2021; Suttle 2022). Although Na, K and Mg were consumed within the adequate ranges, the high intake of Fe and Se at and above H‐400 level exceeded the MTL established by the NRC (2007) for growing lamb groups.

Although sheep can tolerate some mineral excesses, exceeding the MTL for trace minerals like Fe and Se, as observed in this study, poses specific health risks (NRC 2021; Suttle 2022). The elevated Fe intake, particularly above H‐400 levels, is concerning, as excessive dietary Fe is known to antagonistically interfere with the absorption of Cu (de Sousa et al. 2012; López‐Alonso 2012; Spears 2022). This antagonism is critical because Cu was already deficient in the basal diet; thus, high Fe levels may have induced a secondary or functional Cu deficiency. Similarly, excess Se intake can lead to toxicity. Se toxicity in livestock, can manifest as two distinct syndromes: alkali disease (chronic) and blind staggers (subacute), both of which impair animal welfare and productivity (López‐Alonso 2012; Fordyce 2013; NRC 2021). The observed non‐linear intake patterns for several minerals highlight a complex interaction between the *hora* supplement and animal's feeding behaviour, phenomenon also noted by Suttle (2022), where excessive mineral concentrations may trigger physiological feedback mechanisms to limit further ingestion.

### 4.3 Growth Performance and FCE

*Hora* supplementation significantly enhanced growth performance, with the H‐400 group exhibiting the highest LWG, and ADG. This enhanced performance is likely attributed to the combined effects of stimulated appetite (leading to higher CP and ME intake) and the provision of essential minerals in *hora* that support bone and muscle development. Specifically, the daily ME intake of 9.77 MJ/day in the H‐400 group provided sufficient energy density to fuel these significant weight gains. The improved FCE in the H‐400 and H‐500 groups indicates that these levels not only increase DMI but also improved the efficiency of nutrient utilization. This improvement may be driven by highly bioavailable minerals promoting optimal ruminal microbial activity, which in turn enhances fermentation efficiency and subsequent nutrient absorption (Suttle 2022). These findings align with previous reports where mineral supplementation improved ADG and FCE in sheep (Hossein Yazdi et al. 2019; Romo and Boyd 2020).

The significant quadratic polynomial trends observed for all growth parameters strongly reinforce the optimal dose theory. Although *hora* is beneficial up to the H‐400 level, increasing the dose further (H‐500) resulted in diminishing returns. This plateau or slight decline is likely a biological consequence of the functional Cu deficiency induced by the antagonistic concentrations of Fe and Se at the highest supplement levels. As mineral intake exceeds a certain threshold, the metabolic cost of managing mineral excesses may outweigh the nutritional benefits, leading to the observed curvilinear response.

### 4.4 Carcass Characteristics

The improvement in carcass yield largely mirrored the trends observed in growth performance, underscoring the impact of *hora* supplementation on physiological development. The significant increases in EBW, HCW and REA in the H‐400 group highlight the positive impact of *hora* on both total carcass mass and lean meat accretion. These improvements are likely driven by the synergistic effects of increased nutrient intake and the presence of essential trace minerals (Zn, Cu and Mn), which act as critical cofactors for enzymes involved in protein synthesis and muscle fibre hypertrophy (López‐Alonso 2012; Grešáková et al. 2021; Palomares 2022). Our findings align with Spears et al. (2022), who reported improved carcass characteristics in finishing steers supplemented with trace minerals.

The improved HCW and REA in the H‐400 group are particularly noteworthy, as these are key indicators of carcass yield and lean meat content, respectively (Pomar et al. 2009; Yar et al. 2022). The calculated DP was within the expected range and significantly influenced by *hora* supplementation. The DP values are consistent with typical reports for Ethiopian highland sheep breeds (Atsbha et al. 2021) and demonstrate that *hora* supplementation significantly enhances the proportion of saleable carcass relative to the animal's live weight.

### 4.5 Edible Offal Components

The maximum TEO weight at the H‐400 is the most significant findings, suggesting that this level provides an optimal dose of the mineral‐rich *hora* water for enhancing the proportion of edible offal. The observed increase in weights of blood, liver, heart and kidney with *hora* supplementation is a strong indicator of improved metabolic function and organ development. This development is likely a result of the highly bioavailable mineral content of *hora* water, which provides essential mineral cofactors that are known to boost metabolic enzymes, thereby supporting hepatic function and organ tissue development (Killilea and Killilea 2022; Marchetti et al. 2023). Specifically, the higher blood yield can be linked to enhanced intake of Fe and Cu, both of which are vital for erythropoiesis and haemoglobin synthesis (Szabo et al. 2021; Killilea and Killilea 2022). Furthermore, the significant linear increase in tail weight suggests that *hora* supplementation improved overall energy balance and nutrient utilization from the basal diet, facilitating fat deposition in the caudal region.

The observed quadratic trends for TEO, blood, liver and intestine further demonstrate a complex, non‐linear dose‐response relationship. The physiological benefits were not simply proportional to the dose; instead, they peaked at an optimal point (H‐400) and subsequently plateaued or diminished at the highest level (H‐500). This curvilinear response is a crucial finding, reinforcing the biological principle that excessive mineral supplementation can lead to diminishing returns or metabolic stress, as the body redirects energy toward maintaining mineral homeostasis rather than tissue growth (Shokrollahi et al. 2025).

### 4.6 Non‐Edible Offal Components

The highest non‐edible offal components at H‐400 parallel the edible offal findings, indicating optimal overall body structural development and nutrient utilization at this specific dose. This aligns with research highlighting the critical role of adequate mineral intake for optimal growth, reproduction and physiological performance in ruminants (Kegley et al. 2016; Weiss and Hansen 2024). Testicular weight improved with optimal levels of *hora* supplementation, supporting the general positive role of minerals like Zn and Cu on reproductive development. Conversely, penis weight showed an inverse relationship and a strong quadratic contrast, sharply declining compared to the control group. The inverse relationship is contrary to previous studies that generally demonstrated a positive impact of minerals like Zn and Cu on reproductive performance, including testicular growth, semen quality and hormone synthesis (Mayasula et al. 2021; El‐Sherbiny et al. 2022). This complex response suggests that certain mineral components in *hora*, at high concentrations, may exert a localized antagonistic effect on penile tissue development, a phenomenon that warrants further specialized investigation.

The increased spleen and pancreas weights reflect the influence of *hora* minerals (Fe, Cu, Zn, Se and Mg) on metabolic function and organ health. Specifically, these minerals support vital processes such as erythrocyte filtration in the spleen and enzymatic synthesis in the pancreas (Malavolta and Mocchegiani 2018; Xiaocong et al. 2025). The heavier urinary bladder in the H‐200 group, coupled with significant polynomial trends, suggests that the supplement altered excretory workload or tissue mass through its influence on electrolyte balance. Likewise, the highly significant trends observed for the pancreas and the gallbladder (including bile) imply that the *hora* supplement enhanced metabolic secretions, with Zn, Cu and Se likely serving to protect these organs from oxidative stress (Cikim et al. 2023). Although our study demonstrates a positive effect of *hora* mineral water on growth and carcass characteristics, the absence of data regarding blood mineral profiles and organ mineral sequestration is a limitation. Future research should prioritize measuring these parameters to provide direct evidence of mineral absorption rates and the underlying biochemical mechanisms at play in indigenous sheep breeds.

## 5 Conclusion

The study showed that *hora* mineral water supplementation significantly enhances nutrient intake, growth performance and carcass characteristics in growing sheep through a distinct non‐linear, quadratic dose‐response. Supplementation at 400 mL/day was identified as the optimal level, yielding superior results in average daily gain, HCW and REA. Critically, the observed curvilinear relationship characterized by a localized depression in performance at the 300 mL/day level followed by a peak at 400 mL/day highlights the complex interplay between mineral concentration, palatability and metabolic utilization. These findings suggest that exceeding the 400 mL/day threshold leads to diminishing returns. Therefore, providing *hora* at 400 mL/day represents a promising and practical strategy to improve sheep productivity in mineral‐deficient regions. Future research is warranted to explore the effects of *hora* on meat quality, blood mineral profiles and long‐term health to fully elucidate the physiological mechanisms underpinning these production benefits.

## Author Contributions

Conceptualization: Ashenafi Miresa Kenea. Methodology: Ashenafi Miresa Kenea, Taye Tolemariam, Belay Duguma, Ellen S. Dierenfeld, Abebe Nigussie, Feyissa Begna, and Sisay Bekele. Investigation: all authors. Data curation: all authors. Writing – original draft preparation: all authors. Writing – review and editing: all authors. Supervision: all authors. All authors have read and agreed to the published version of the manuscript.

## Funding

This research was supported by Jimma University College of Agriculture and Veterinary Medicine (Grant number AgVmVm/M6/23) which provided funding for data collection and laboratory analysis.

## Ethics Statement

This study was performed in line with the guidelines of the European Union directive number 2010/63/EU (2010) regarding the care and use of animals for experimental and scientific purposes. Approval was granted by the Ethics Committee of Jimma University, College of Agriculture and Veterinary Medicine (RGS/588/2023).

## Conflicts of Interest

The authors declare no conflicts of interest.

## Data Availability

The data that support the findings of this study are available from the corresponding author upon reasonable request.
